# Monopoly on doubt: Post-mortem examinations in Israel, 1950s–1980s

**DOI:** 10.1093/shm/hkad101

**Published:** 2024-01-13

**Authors:** Benny Nuriely, Liat Kozma

**Affiliations:** Independent Scholar; Department of Islamic and Middle Eastern Studies, Faculty of Humanities, The Hebrew University, Jerusalem, Israel

**Keywords:** post-mortem examination, biopolitics, civil protest, Israel

## Abstract

This article examines the durability of high post-mortem examination rates in Israel between the 1950s-1980s. Previous studies overlooked the issue of medical authority and the social history of autopsy, focusing on policy, technological development, and conflict between science and religion. By contrast, our analysis brings together the medical interest in unlimited research of dead bodies and the power relations between doctors and subaltern groups in Israel. Based on the Israeli State Archives, the Hebrew University Archives, and the daily press, we argue that medical biopolitical aspirations and the public shaped the history of postmortem examinations in Israel. High rates were embedded in the medical construction of doubt regarding the cause of death that only physicians could resolve by autopsy. Civilian protests led to a temporary decrease in the 1960s, while political and medical intervention brought about a gradual resurgence in post-mortem rates in the 1980s.

In April 1965, several families who had immigrated to Israel from Mashhad, Iran, filed a lawsuit against the Greenberg Pathological Institute in Tel Aviv. A few days earlier, seven members of the Mashhad community died in a car accident.^1^ Since the cause of death was known, the Minister of Police assured them that no post-mortem examination would be performed. Nevertheless, one was conducted. In addition to the suit, the families had 25,000 citizens sign a petition, calling ‘to end lawlessness in postmortem examinations [...] there was no legal and humane reason to abuse the bodies of our loved ones’.^2^

The Mashhad 7 protest was part of a broader social resistance to post-mortem examinations (PMEs) in Israeli hospitals, which included demonstrations, public petitions, letters of complaint, and even riots. The Israeli 1953 Anatomy and Pathology Law permitted PMEs only in case of doubt regarding the cause of death. In practice, however, doctors insisted that the cause of death could never be known with absolute certainty and performed PMEs on most bodies reaching Israeli hospitals—at rates that reached 70–90 per cent in some hospitals by the late 1950s (see Figure 1). ‘Doubt’ thus enabled nearly unlimited access to corpses, an access which doctors explicitly prided on since, in their view, it placed Israeli medical science at the forefront of global scientific research.

**Fig. 1:**
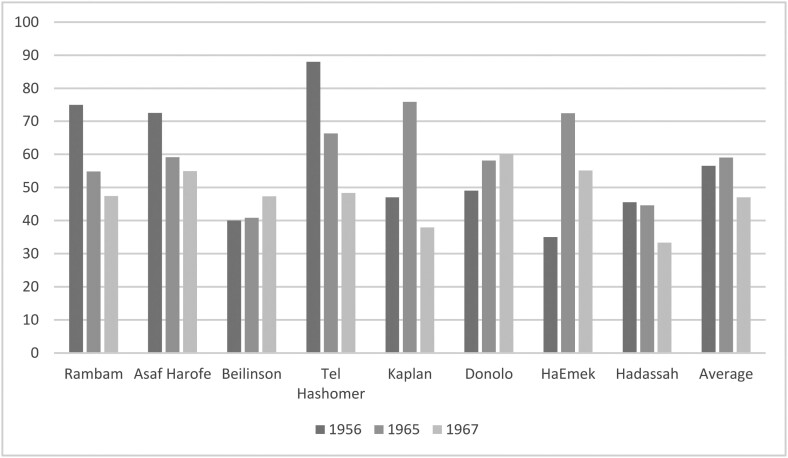
Post-mortem examination (PME) rates by hospital in the years 1956, 1965 and 1967

This article examines the high PME rates in Israel (compared to Western Europe and the USA), the constitution of ‘doubt’ by the medical profession as a justification for PME routinisation and attempts to challenge that state of affairs. We start with the legislation of the Anatomy and Pathology Law and end with the consequences of the amendments to the law in the 1980s. To explain the durability of high PME rates, our analysis brings together the medical profession’s interest in an unlimited research laboratory of dead bodies and the relations between doctors, the political system and the religiously and culturally diverse Israeli public.

The uniqueness of the Israeli case, we demonstrate, lies in two aspects. First, the Jewish taboos over delayed burial and damaging the body’s integrity clashed with doctors’ insistence on PME.^3^ Second, doctors’ employment of ‘doubt as to the cause of death’, as articulated in the letter of the law, in order to justify the performance of PMEs on most dead bodies in Israeli hospitals. The Anatomy and Pathology Law empowered the medical profession and its professional aspirations, prioritising medical research and training over patients’ informed consent.

PMEs became a routine part of medical practice in Western Europe in the second half of the 19th century due to the expansion of public hospitals and hospital-based scientific research.^4^ Toward the mid-20th century, high PME rates became a key factor in hospital accreditation.^5^ In the 1940s, rates peaked at more than 40 per cent and remained stable for two decades.^6^ By the late 1960s, they dropped below 2.5 per cent in China, and in the early 1970s and 1980s, they dropped below 20 per cent and 10 per cent in Europe and the USA, respectively.^7^ Two main factors explain this decline: medical authorities cancelled the minimum rates for hospital accreditation in the early 1970s, and imaging technologies enabled more accurate diagnoses of living patients.^8^

Studies on PME in Israel have concentrated on the struggle between religious and medical authorities. Legal scholars have argued that the political Jewish-religious protest of PMEs, led by the ultraorthodox party Agudat Israel (AI) and the National Religious Party (NRP), was an instrumental one and served, for example, as a bargaining chip in the religious parties’ entry into the ruling coalition.^9^ This scholarly focus on the clash between religion and science misses out, first, the fact that most protesters against PMEs were not members of ultraorthodox or national-religious parties or groups, but underprivileged immigrants from Arab and Islamic countries. Second, this body of scholarship tends to ignore the social and political power of the medical profession, and the active role it played in the broad legitimisation and routinisation of PME.

Our research relies on the Israeli State Archives (ISA) and the daily press and makes the following arguments. First, high PME rates relied on the monopolisation of doubt regarding the cause of death—a doubt that only medical authorities could resolve and only by resorting to PMEs. Initially planted within the letter of the law, this doubt enabled Israeli physicians virtually unhindered access to dead bodies for scientific research. Following Michel Foucault, we analyse the Anatomy and Pathology Law as an effect of biopolitical practices.^10^

Foucault defined biopolitics as population management, a set of practices aimed at controlling births, deaths, diseases and labour productivity. It includes the production of statistical data and various medical, juridical, police and educational practices. Foucault excludes the dead from his definition of biopolitics: ‘Death is outside the power relationship. Death is beyond the reach of power’.^11^ However, in our case, population management included the dead as well, as a population and as a raw material for scientific research. The value of scientific knowledge that the PME produces, that pathologists appropriate, and that serves the medical profession also requires the management of the dead. In the case of PMEs, the medical profession has a relationship of dependency and appropriation with dead bodies, which become a means of knowledge production. This knowledge production, in turn, required the medical profession to struggle against individuals, families and communities and mobilised for this purpose the political system, the police and the press. The formulation of the law, its implementation and the routinisation of doubt in hospitals vis-à-vis the political system and relatives of the deceased constituted doctors’ professional authority to dissect bodies for research, thus transforming Israeli hospitals into pathological laboratories.

Second, building on Khaled Fahmy’s observations regarding how non-elites in 19th-century Egypt constructed their own understanding of forensic autopsies and utilised its mechanisms,^12^ we argue that Jewish immigrants and ultraorthodox groups had a keen understanding of PME, its purposes and the law. Turning first to parliament members and the press, they later held demonstrations demanding that bodies would be dissected only upon informed consent. We argue that reducing the conflict over PME to religion vs. science ignores the ethno-class power relations between the medical profession and the public, which shaped the history of PME in Israel. We thus add to the discussion social actors hitherto silenced in research—underprivileged families and communities who protested the misapplication of the law.

Third, we argue that civilian protest against PME is associated with fluctuations in PME rates throughout the period under study. Overall, during the period under study, PME fluctuated between 56 per cent in 1956 and 59 per cent in 1965, fell to a temporary low of 43 per cent in 1973 and then rose again to 59 per cent by 1989.^13^

The remainder of this article is composed of three sections. The first examines the background of the 1953 Anatomy and Pathology Law and its aftermath, as doubt was implanted within the letter of the law. The second analyses the increase in PME rates, followed by widespread protests, including public petitions, letters of complaint addressed to parliament members, lawsuits and demonstrations. Their struggle was followed by a temporary decline in PME rates and physicians’ attempts to mobilise the political system to preserve their monopoly over the decision to perform PMEs. The last section explores the growing political discontent over PMEs in the 1970s, resulting in the amendments to the law in 1980, followed by a gradual resurgence in PME rates.

## The Anatomy and Pathology Law: Framing and Implementation, 1951–60

The 1953 Anatomy and Pathology Law addressed two types of autopsies. First, dissection for the study of anatomy at the Hebrew University Medical School, the only one in Israel at the time, in cases in which citizens have donated their bodies for this purpose and in cases where the deceased had no one (neither next of kin nor a religious burial society) who demanded their bodies and immediate burial. Second, PME in non-criminal cases was permitted when the cause of death was unknown or to use organs to heal patients—subject to a certificate signed by three doctors authorised for this purpose by the Ministry of Health. Forensic dissection was regulated in the Cause of Death Investigation Law of 1958 and is beyond this article’s scope.

There are three main differences between anatomical dissection and PME. The former is conducted for teaching at the medical school, while the latter is conducted in hospitals. Second, anatomical dissection is a yearlong process of stripping the body to the bone, while PME concentrates on the pathology that killed the patient and the body returns to the family for burial within a day or two. Third, anatomical dissection does not require the patient’s case history, while PMEs rely on patients’ case histories and compare clinical and post-mortem findings.^14^ The pathology part of the law had much broader implications than the anatomy one: whereas the medical school relied on 30 bodies per year, pathological autopsies were conducted on most bodies of those who died in Israeli hospitals.

The first agreement on PME was signed as early as 1944 between the Chief Rabbinate of the Jewish community in Palestine and Hadassah Hospital. The arrangement stated that examinations would be performed on those who explicitly permitted it in cases where the discovery of the cause of death could prevent the death of others (e.g. in case of hereditary disease or epidemic) and in cases where it could not be determined otherwise. Such authorisation would have to be signed by three doctors.

This agreement constituted a part of a broader arrangement between the secular Zionist elite and the Jewish-religious establishment. In the 1940s, the Zionist leaders sought to produce a broad Jewish consensus toward transforming into a future state. This process culminated in the Status Quo pact signed in 1947 between the heads of the dominant party (Mapai, Workers’ Party of the Land of Israel) and religious leaders. The pact offered the religious leaders control over the Ministry of Religion, matrimonial arrangements (marriage and divorce among Jews) and the observance of Saturday in state institutions.^15^

The Status Quo pact indicated mutual dependency in the Jewish society in Palestine. Religious groups depended on the secular elite, who managed the Jewish society economically and militarily, while the secular elite strove to create a Jewish state, hence, needing the legitimation of the Jewish-religious leadership. After establishing the Jewish state, the two have been struggling over the identity of the new state. The secular elite advanced technological and scientific development, freed from religious sensibilities.^16^ Religious parties, on their part, held their monopolies and sought to introduce more Jewish elements into state mechanisms.^17^

In January 1951, Israel’s Chief Rabbinate complained to the Minister of Health about the high rates of PME in Israeli hospitals, which included cases in which there was ‘no doubt as to the cause of death’. General Director of the Ministry of Health Chaim Sheba, whose policies in subsequent years as Director of Tel Hashomer Hospital we discuss below, argued that only a PME could determine the cause of death—and that doubt always exists. Sheba explicitly argued that the main reason for conducting PMEs had little to do with individual patients’ well-being but that it was crucial for doctors’ professional development and saving lives. PMEs contribute to the international reputation of Israeli medicine; without them, wealthy patients would resort to private hospitals abroad.^18^

The motivation for a broad definition of doubt was, therefore, explicit—maintaining the professional level of practicing physicians and improving Israeli medicine. Sheba framed a broad definition of doubt to justify the broadest possible authorisation for PMEs, which he deemed essential for the professional level of Israeli medicine. This framing prioritised scientific progress over persons’ control on their own dead bodies and that of their loved ones. In a similar vein, the preamble to the draft law, formulated in 1951, explained that ‘PME, in case of doubt regarding the cause of death, is vital for doctors’ professional improvement and progress, and thus serves the well-being of patients and improves their likelihood of recovery’.^19^ This justification disappeared in the final version of the law, thus erasing this crucial motivation for its legislation.

In the deliberations leading to that version, religious party members maintained that the requirement for the consent of three doctors was bogus since doctors signed all PME requests automatically. In the deliberations of July 1953, Parliament Member Kalman Kahana (AI) suggested that one of the three doctors be a government doctor nominated for this purpose, who would also consult a religious authority of the community to which the deceased was affiliated. Parliament Member Zalman Ben Yaakov (AI) similarly suggested that one doctor would be appointed by the Ministry of Health, one by the deceased’s family, and one by their religious community.^20^ In August, Deputy Minister of Religions Zerach Varhaftig of the National-Religious Party (NRP) suggested that the family appoint one of the three doctors. All three suggestions were rejected.^21^

The final wording of the law created the loophole that would be at the heart of the controversy for three decades: ‘A doctor may dissect a dead body to determine the cause of death or to use part of it to heal another person if such a dissection has been approved in a certificate signed by three doctors appointed for this purpose in the regulations’.^22^ As the law nominated members of the medical profession exclusively as arbiters of doubt over the cause of death, more than two dozen government hospitals and sanatariums became a national laboratory that provided almost unlimited access to corpses.

In the following years, citizens and community representatives sent complaint letters to ministers, parliament members and the press about PMEs performed without the family’s knowledge and despite their objections. Physicians interpreted the permission to determine the cause of death in the broadest sense possible—taking the liberty to conduct PMEs in virtually every case. In 1954, a citizen from a small and remote town published a piece about two PMEs performed despite explicit family opposition. One of the bodies released to burial was operated on ‘from head to toe’.^23^ Similarly, an immigrant in a transit camp wrote to the Ministry of Health that ‘each and every dead person is being operated on here, something unheard of in other countries where only unidentified bodies are operated on. The humiliation of the dead of Israel cries out to heaven’.^24^ In another case, a rabbi wrote a letter to the Minister of Religions, stating that:

Doctors sent some patients to Tel Hashomer Hospital. They died 2–3 days later, and their bodies are returned operated on without informing their relatives. We believe you can prevent such degrading acts, which are against the law and lead to public resentment.^25^

Both letters emphasised the importance of the (Jewish) honouring the sanctity of the human corpse. However, alongside religious sentiment, the letters also highlight the violation of civil rights, namely the denial of citizens’ right to take part in determining the fate of their own bodies or that of their loved ones. Deputy Minister of Religions Varhaftig made a similar argument in his letter to Minister of Health Yosef Serlin in early 1954. Varhaftig explained that PME rates were extremely high, claiming that the required authorisation of three doctors ‘became a mere formality’. He added that this situation was ‘complete lawlessness’ and pleaded to enforce the law and punish the perpetrators.^26^

Serlin vehemently denied Varhaftig’s claim, arguing that PMEs were performed according to ‘meticulous indications’ under the supervision of hospital directors. PMEs, he emphasised, were performed when doctors failed to make a diagnosis, when the patient died shortly after arriving at the hospital, or when the diagnosis had been established, but the treatment failed. In such cases, PME was conducted to benefit other patients with similar diseases and treatments. Besides, doctors were obliged to record the cause of death and could not do so when it was unknown.^27^ Here the Minister insisted that PMEs were the exception, despite allegations that they were the rule.

Dr. Rafael Gjebin, Director of the Health Services Division at the Ministry of Health, on his part, demanded that the professional level of Israeli medicine be prioritised over citizens’ concerns with PME. To him, PME was a professional practice of national importance, which set Israel apart from its neighbours and placed it at the same level as the most developed nations: ‘Israel can take pride in its high medical level, which not only exceeds the level of the Levantine countries but can compete with enlightened and advanced countries’. Gjebin suggested that citizens donate their deceased relatives to improve Israeli medical science. Otherwise, excellent doctors would leave the country, and medicine would deteriorate to ‘Levantine’ standards.^28^

Varhaftig wrote again to the Minister of Health and the Attorney General, this time about a 71-year-old cardiac patient who objected to his PME to the extent that he took the trouble of sticking a note into his ID card demanding not to operate him after death. Varhaftig noted further PMEs conducted on elderly patients, suicide cases and drowning victims, namely cases in which there was no doubt regarding the cause of death. He asked again that the law be respected and PME restricted to cases where the cause of death was unknown. PME rates are too high, he emphasised, stressing that the cause of death could be known in the vast majority of cases.^29^

Following Varhaftig’s complaints, the Minister of Health appointed a commission of inquiry.^30^ Two doctors were appointed to head the commission, Dr. Mordechai Meir from the Ministry of Health and Dr. Friedenthal from Donolo Hospital in Jaffa. They were required to examine whether PMEs were performed according to the law, whether each PME was ratified with the signature of three authorised doctors, and whether the manner of closing the body after the PME was respectful.^31^ They concluded that the rate of PMEs was not excessive and that PMEs were being performed according to the law.^32^ Dr. Meir argued that a comparison of PME diagnosis with that of a living patient often revealed unforeseen differences; the benefits of PME, both scientific and clinical, were therefore significant. Even when the deceased was elderly, questions about the mechanism of the disease often arose, and resolving them could improve future prevention.^33^ Dr. Meir’s reasoning illustrate how medical practice expanded the definition of doubt regarding the cause of death to legitimize PMEs at the expense of patients’ or their relatives’ informed consent. Doubt thus became the rule rather than the exception.

Despite the committee’s conclusion, public opinion was a concern for doctors and administrators at the Ministry of Health. In late 1955, the same Dr. Meir, who had claimed a year before that PMEs were performed according to the law, wrote to the Director General of the Ministry of Health that pathologists deviated from the criteria specified by law. Dr. Meir explained that the high PME rates in Israel were desirable scientifically but threatened to exceed the limits of the law. He argued that the law’s wording had been achieved with great effort and with the consent of the Chief Rabbinate. News of excessive rates would lead to complaints that might eventually lead to restrictions and even to an undesirable law amendment. Therefore, he suggested instructing pathological department heads to take legal restrictions more seriously.^34^

Dr. Meir was concerned that the liberties doctors were taking might backfire. He offered to reinterpret the law rather than ‘rely on illogical literal interpretations, contrary to the legislature’s intention’. Consequently, the Director General of the Ministry of Health instructed government hospitals in February 1956 to be more cautious. His letter referred to PMEs executed ‘irrespective of the legislator’s intention, and in ways that, according to complainants, make a mockery of the law’. He demanded limiting PMEs to cases in which three doctors believed the cause of death could not be explained otherwise. He wrote that PME rates of 60–80 per cent ‘contradict the law that regards autopsy as an exception. That could lead to more complaints and pressures to change the law’.^35^

In 1957, a Ministry of Health survey suggested that the national average in government hospitals was 60 per cent, reaching 93 per cent at Tel Hashomer.^36^ A few years later, a survey conducted at Tel Hashomer found that PME rates in 1961–62 were 73 per cent. The highest rate was among infants, 92 per cent. Of these, the highest rates were among Jews of North African descent (100 per cent), Jews of Asian descent and Palestinian citizens of Israel (93 per cent). The report noted that in a similar survey conducted on a multi-ethnic population in the USA, PME rates among Jews were 36.8 per cent, while among Christians, it ranged from 60 to 70 per cent.^37^ The mention of the survey conducted in the USA did not include a reference, and we cannot verify its reliability. However, recent research shows that in the 1960s, the average PME rates in the USA were 40 per cent and began declining in the early 1970s.^38^

The exceptionally high figures at Tel Hashomer Hospital, founded in 1953, were not coincidental. According to Nurit Kirsh and Ari Barel, the hospital’s first director, Dr. Chaim Sheba, used it as a laboratory of sorts, as its doctors had access to a large and genetically diverse population and control and manipulation of research subjects. Due to Sheba’s earlier positions in the Israeli health system, as the founder and first commander of the Israeli Medical Corps (1948–50) and later the General Manager of the Ministry of Health (1950–52), Sheba had close ties with Israel’s political and military elites and thus significant political impact. Sheba took particular interest in Jews from Arab and Islamic countries. Their vulnerability to medical research as a marginalised population, large families that enabled multigenerational genetic mapping and presumed high consanguinity rates made them ideal subjects. It is, therefore, not surprising that Sheba came to consider almost universal PMEs as an acceptable practice.^39^

In May 1959, ultraorthodox parliament members complained to the Minister of Health that hospitals performed PMEs on people who had died of natural causes against their family’s will, claiming that ‘the cause of death is unknown’.^40^ More complaints came from Jewish and Muslim clerics who argued that elderly people in nursing homes were taken to hospitals before dying to undergo a PME.^41^ Consequently, religious Jewish and Muslim patients started avoiding hospitals.^42^

Again, the Director General of the Ministry of Health instructed to restrict PMEs to the legally stipulated criteria,^43^ and again pathologists argued that PMEs were medically necessary. ‘In its current form, the law permits PME of all bodies’,^44^ they insisted and claimed that these operations were critical for scientific progress.^45^ Public complaints, administrative instructions to curtail PMEs and in response—hospitals’ medical justifications recurred in the 1950s.^46^ In the following years, complaints to parliament members and the press only increased, and resistance to PMEs included tens of thousands of protesters who marched in the streets and clashed with doctors and the police. Civil protests sometimes escalated to violent riots, incorporating additional actors who joined the struggle over PMEs: the legal system and the Israeli Medical Association.

## Escalation: The Popular Struggle Against the Medical Establishment, 1960–67

In the early 1960s, PME rates increased, and complaints about PMEs performed without prior notice or consent intensified. After two failed attempts to amend the law, the protest became massive, including petitions signed by tens of thousands and violent attacks on medical personnel. The rise in PME rates, on the one hand, and failed attempts to amend the law, on the other, brought about civil protest. Immigrants from Arab and Islamic countries—the prime target of this medical practice—and ultraorthodox Jews of European descent held mass demonstrations. The protest culminated in 1967, followed by a considerable yet temporary decrease in PMEs nationwide.

In January 1961, a man whose mother had died of cancer accused Hadassah Hospital in Jerusalem of removing her organs: ‘Are patients who come to heal their pains in Hadassah abandoned? This opposes morality, justice, and our holy religion and violates the law since the organs removed are not used to heal another person’. The doctor replied that the mother had suffered from many illnesses that could not explain the symptoms that led to her death, and since the actual cause of her death was unknown, her PME was legal, and its findings were crucial for the diagnosis of future cases.^47^

In February, a lawyer wrote the director of Hadassah Hospital. A dying patient came to the hospital with his daughter and son-in-law. After the latter returned from a short break, the doorkeeper in the ward told them that he had received an explicit instruction to prevent their entry. Through the window, they saw a nurse covering the deceased with a white sheet. Then a doctor approached and told them that the body would not be operated on since the cause of death was known. However, when representatives of the burial association arrived the next day to collect the body, they found that ‘the head was open from ear to ear, a hole was also found in the back, the body was cut from the neck down, coarse seam’. The family insisted for hours that all organs be returned to them, and their lawyer demanded a criminal investigation.^48^ In June, a man complained to the Ministry of Religions that his daughter, who had died of a long illness, was operated on despite an explicit promise by the doctor at Tel Hashomer. The director of the paediatric ward replied that it was not clear whether the cause of death was ‘a cancerous process, degenerative disease or poisoning’, and therefore, PME was needed.^49^

In this period, resistance to PMEs was expressed mainly through complaint letters to members of the religious parties, the NRP and AI. The two parties initiated two failed attempts to amend the law. In 1961, the Israel Medical Association (IMA) stepped in and warned that the proposed amendment would harm public health, hamper medical research and damage national medical education. Again, the doctors did not claim they were abiding by the legal restrictions, but rather that routine PME benefited scientific progress.

Our medical achievements since the establishment of the state in diagnosing diseases, treating patients, medical research, raising our profile among the world’s scientific community, and educating a generation of Hebrew doctors would be severely disrupted.^50^

From 1965 to 1966, amending the law was discussed again, and hospital directors and the IMA were again quick to respond. IMA representatives stated that the amendments would lower PME rates and undermine medicine. IMA’s intervention was aimed to hamper the attempts to amend the law, arguing that the 1953 law was adequate to the satisfaction of all parties. For example, ‘New drugs have side effects, and only PME can reveal their medical worth’.^51^ Furthermore, ‘in large hospitals’ in most European countries, PME rates reach 100 per cent; in Western Europe, it ranges from 75 to 95 per cent. In addition, ‘in the major states of the USA’, PMEs are decided by a state-authorised ‘medical examiner’. In any case, they concluded, PMEs must depend solely on medical, not political considerations.^52^ Labor Minister Yigal Alon agreed, claiming that ‘after all, doctors are the experts on this subject’.^53^

The IMA’s response coincided with growing resistance to PME by Jewish immigrants from Arab and Islamic countries. One conspicuous event was the Mashhad 7 case which opened this article. Another came to be known as the Revaha-Kaplan Case. In May 1966, a violent incident occurred at Kaplan Hospital in Rehovot, near some immigrant agricultural communities. The body of a 70-year-old man from Revaha, a village of immigrants from Kurdistan, was brought to the hospital. Despite a guarantee the hospital had given the family, the body was dissected, its internal organs removed and its eyes replaced with glass buttons. The dispute between the family of the deceased and doctors escalated quickly into a fight that ended with five villagers, four doctors and five police officers injured. The family demanded to investigate the hospital and that the police return the deceased’s organs. The hospital responded, ‘The doctor who signed the death certificate did not know that the body was operated on’.^54^ It decided to collectively punish all residents of Revaha and deny them medical treatment until they apologised. The following day, three patients from Revaha were denied treatment; two of them returned home and a third was transferred to another hospital.^55^

A week later, the incident was discussed in parliament. The Minister of Health defended Kaplan. He condemned the villagers for their violence against ‘law-abiding’ doctors. He also criticised the hospital’s decision to deny medical treatment ‘in response to a riot, and even though the deceased was operated on according to the law’.^56^ The hospital management feared a lawsuit and invited a Revaha representative the following day. The representative apologised for the incident on behalf of the village, and the hospital expressed regret over the frustration caused to the deceased’s family ‘due to a mistake made by one of the doctors’. The representative asked that no more bodies be operated on without consent, and the hospital management gave an oral, but not a written, promise to that effect.^57^ A few days later, the police filed an indictment against 15 residents of Revaha for assaulting doctors and police officers and participating in a riot. The trial took place a few months later. The prosecution argued that ‘these are primitive and violent people. Therefore, we should not conclude that they understand the great controversy on PMEs’. The judges accepted the prosecution’s claims and argued that ‘the court’s position is that the problem of autopsy should not be discussed’ but insisted that ‘the defendants’ actions were grave’.^58^ The court sentenced six of the accused to 3 months to a year in prison.

The prosecution, followed by the court, framed them as an ‘incited’ ‘oriental’ mass ignorant of the meaning of its own protest.^59^ As the struggle against the medical profession swept the Jewish lower classes, the monopolisation of the dead required dismantling the volatile ethno-class tension, in this case, between the medical profession and underprivileged migrants. The court attempted to neutralise this explosive issue by labelling the protesters as uncivilised and unworthy of participating in public discourse. The media framing of this incident further denied the agency of most protesters by focussing on two groups: ultraorthodox Jews and doctors—both predominantly of European descent. This framing was later reproduced by historians, sociologists and legal scholars using the binary distinction between scientific ‘rationality’ and religious ‘irrationality’.^60^

The Revaha incident marked the beginning of a mass protest, which led to the establishment of an anti-Zionist ultraorthodox organisation: the Committee for Safeguarding Human Dignity (CSHD). They organised rallies, marched on the streets and clashed with the police, interrupted hospital work, wrote petitions to politicians and hospital directors and disseminated pamphlets shaming pathologists. Together with the activities of this group, anti-PME activism peaked in 1967 and continued until the mid-1970s. In August 1966, 2,000 members of the Yemenite community demonstrated in Rosh HaAyin and 3,000 ultraorthodox Jews demonstrated in Jerusalem.^61^ In February 1967, thousands blocked streets and clashed with the police in HaTikva, a Tel Aviv neighbourhood of working-class immigrants from Arab and Islamic countries.^62^ A month later, members of Yemenite communities living in Rehovot and Rishon LeZion held a demonstration near Kaplan Hospital.^63^ A week later, 3,000 ultraorthodox demonstrators blocked several streets in Jerusalem and threw stones at the police; dozens of police officers were injured and 17 were arrested.^64^ Throughout 1967, dozens of letters were sent to hospitals nationwide, demanding not to perform PME on relatives.^65^

No evidence indicates concrete connections between immigrants from Arab and Islamic countries and the ultraorthodox protesters. Both groups were economically and culturally marginal. We may assume that they both shared Jewish sentiments regarding the dead. An essential element they shared was the demand that doctors should perform a PME on their loved ones only after obtaining informed consent. In other words, sociological marginality and demand to preserve civil rights brought together the immigrants and ultraorthodox in a specific historical moment.

The following years witnessed a temporary decline in PME rates. A 1975 study found that the national average of PMEs in governmental hospitals rose from 56.5 per cent in 1956 to 59 per cent in 1965 and dropped to 49 per cent in 1967, at the height of the protest. Kaplan Hospital experienced the sharpest decline, from 75.9 per cent in 1965 to 37.9 per cent in 1967. During the same years, other significant decreases occurred at Rambam, from 72.4 to 55.1 per cent and Tel Hashomer, from 66.3 to 48.3 per cent.^66^

Not all hospital directors objected to this trend. In April 1967, Shaare Zedek Hospital in Jerusalem issued a statement supporting a law amendment. Serving mainly the ultraorthodox community in Jerusalem, it supported a compromise.

The rights of the family should not be ignored […]. It is customary in all enlightened countries to consider the family’s opinion, and in some countries (e.g., the United States), written consent of family members is required. Performing a PME without such consent is considered a criminal offense.^67^

This announcement was unique and did not reflect the mainstream of the medical profession that invested tremendous efforts in defense of the monopoly on doubt. For example, in April 1967, IMA Chair Prof. Emil Adler convened a meeting with the directors of government hospitals that served as teaching hospitals. The meeting was designed to discuss ways to counter the opposition to scientific research on corpses.^68^As seen below, a year later, the minutes of this meeting reached ultraorthodox Parliament Member Shlomo Lorincz, who disclosed it in parliament.

The ultraorthodox protest against PMEs described above was well-organised in terms of civil activism and parliamentary support, and the medical establishment had to invest more substantial efforts in dealing with their demands. This was not the case with most protesters, migrants from Arab and Islamic countries, whose complaints were largely ignored. One example of this gap is the case of a rabbi’s wife whose heart was removed in Tel Hashomer without her family’s consent or the signatures of three authorised doctors. In contrast to the Mashhad 7 and Revaha-Kaplan cases, the rabbi’s wife case led to a statutory commission of inquiry that concluded: ‘It was a mistake to remove the heart of the deceased without the signatures of three doctors and it is regrettable that no explicit instructions were given to the doctors who treated the patient in her last hours’.^69^ The Ministry of Health Deputy Director asked Dr. Sheba for his response. The latter refuted the very suspicion of his doctors’ integrity. He claimed the removal of a heart was not considered a PME, and the absence of signatures by three doctors was a ‘mere technicality’, indicating that ‘we did not take the easy way of preparing such a certificate to cover up our job’.^70^ Sheba argued that his pathologists should be appreciated for not concealing the operation.

Furthermore, as protests escalated in the 1960s, doctors encountered objections to PMEs in hospitals and tried to mobilise the political system to regain their hold over PMEs. In August 1967, Sheba wrote, ‘The public became used to refusing PMEs and threatening us. The police give burial certificates even if we write that the cause of death is unclear. They should be instructed not to give a burial certificate’.^71^ A few months later, a pathologist from Tel Hashomer complained to the Minister of Health about difficulties they had faced when he requested the removal of the eyes from the deceased due to opposition from religious circles. Director General of the Ministry Gjebin claimed that ‘when you ask families of the deceased to use the cornea, you are refused in many cases. If you do not ask the families, you get violent reactions and scandals’.^72^ In early 1968, IMA representatives appealed to the Prime Minister, the Minister of Justice and the Minister of Health to restrain the CSHD: ‘They have crossed all boundaries; they incite against doctors, and all attempts to appease them have failed. The government must protect doctors and restrain these protesters’.

In the following years, civil pressure led to political interventions that revealed a widespread medical phenomenon: since the law was enacted in 1953, doctors treated the three-signature requirement as a mere formality. Doctors pre-signed PME certificate forms, and the names of the deceased who operated on were added retrospectively. This phenomenon was termed ‘blanco [blank] forms’.

## Managing Pandora’s Box, 1968–80

Public discontent with PMEs and questioning doctors’ motivations became widespread following the popular protests of 1967. They were expressed in parliament, first with the publication of the minutes of the April 1967 meeting and then with the publication of the State Comptroller’s annual report in early 1969. In the 1980s, following a change of government, the Anatomy and Pathology Law was amended to add patients’ and families’ consent as a requirement for a PME. However, following the amendment, the national average of PME rates rose rather than decreased.

In November 1968, Parliament Member Lorincz of the AI presented the minutes of the April 1967 meeting of hospital directors,^73^ which indicated that doctors had been violating the law for years. Prof. Adler, who summoned the meeting, argued that it was impossible to teach students^74^ without PME and that this was the responsibility of the teaching hospitals. Dr. Sheba saw this debate as a war: he demanded that minimum PME rates be set, as in other countries such as the USA, and that hospitals that would not meet the quota would not be qualified to teach students. ‘This would push our institutions to go to war’.

Dr. Eliezer Geltner, Director of Assaf Harofeh Hospital, noted that PME rates of over 60 per cent at his hospital were achieved using patients from the geriatric department and by concealing PMEs from families: ‘We do not ask the families, we take the body and operate’. Sheba concurred, claiming that ‘we literally steal the PME from families in various ways’. Dr. Bezalel Naor, director of Kaplan Hospital, claimed that to prevent resistance, ‘an order was not to indicate on the death certificate whether PME was performed, because the certificate gets into the hands of the families’. Towards the end of the meeting, Sheba suggested an ambitious plan: allocating separate hospitals to those who oppose PMEs, alongside ongoing pressure on the political system. Adler concluded the meeting with a proposal to pressure teaching hospitals to meet a future PME quota. After quoting from the minutes, Lorincz demanded a parliamentary committee of inquiry to investigate the meeting's participants. However, Minister of Health Israel Barzilai rejected his proposal, arguing that the minutes merely expressed an ‘exchange of opinions without any decision, and without violating the law’.

In early 1969, however, the State Comptroller published his annual report,^75^ which intensified public criticism of the medical establishment. The report presented partial data on 5 out of 12 government hospitals in which PMEs were performed in 1965–68. Most hospitals refused or evaded requests to present data.^76^ According to the report, in 1965, 2,264 PMEs were performed out of 4,113 deaths (55 per cent); in 1966, 2,314 PMEs were performed out of 4,747 deaths (49 per cent); and in 1967, 2,174 PMEs were performed out of 4,873 deaths (44 per cent). On the other hand, it was claimed that in 1967, only 1,756 PMEs were performed in the five hospitals (418 less than previously claimed), of which 424 (24.1 per cent) were conducted without the required authorizations: 307 PMEs were signed for by only two physicians, 53 by one and 64 with no signature at all. In January–October 1968, 1590 PMEs were performed, of which 450 (28.3 per cent) were without the required authorizations: 336 under the signature of two physicians, 48 under the signature of one and 66 without any. The report concluded that the Ministry of Health failed to enforce the law and recommended supervision arrangements for hospitals by the Ministry.

Despite the partial data presented, the State Comptroller’s report opened Pandora’s box that the medical establishment and government ministers tried to close for a decade and a half. Many PMEs have been performed under pre-signed ‘blanco forms’. The opening of Pandora’s box revealed that the critics who claimed in the early 1950s that the law was a mere formality were right. Less than three doctors signed many PMEs, and as we shall see below, in many other cases, PME forms were signed in advance and kept for future use. In July 1969, Israel’s Attorney General wrote the Minister of Health that the state lacked the constitutional ability to supervise PMEs. Instead, he suggested asking the State Comptroller for specific data on which his report was based.^77^

At this stage, the Ministry of Health took it upon itself to manage the Pandora’s box. First, the Minister sought to assuage complaints by religious parliament members. Backed by the annual State Comptroller report, the Minister of Religions, on his part, demanded a commission of inquiry to examine delinquent doctors who used corpses for research when the cause of death was ‘known even to a layperson, such as death by train accident’.^78^ A month later, the Minister of Health instructed all hospitals to send lists of doctors authorised to sign PMEs ‘to make the Minister of Religions’ demand superfluous’.^79^

We hope that a decision on the Anatomy and Pathology Law will be made soon. The State Comptroller is pressuring—and he is not the only one [...] the state attorney stresses that currently there is no status to any demands by the family. Provisions regarding three signatures apply to *all* cases.^80^

Reviewing the reports hospitals sent to the Ministry of Health revealed PMEs performed under the signature of physicians who were not authorised signatories and physicians who performed PMEs without authorisation. In early July 1969, a letter^81^ was sent to the Ministry of Health about a corpse found in a sanatarium in Jerusalem. The death certificate showed that the body was dissected. However, the doctor in charge wrote, ‘No case of dissection was documented [...] neither in July nor for the entire year 1969’. In November 1969, another PME without signed authorisation was reported to the Ministry of Health. Gjebin threatened the Director of Rambam Hospital: ‘Next time, I will bring such a case to the Attorney General to prosecute the doctor who violated the law. All pathologists must know that no PME should be performed before they have a legally signed certificate’.

Some hospitals may have adapted their practice to the instructions of the Ministry of Health. Nevertheless, the data indicate that the medical routines that began in the early 1950s changed little, relying on the same rationalizations. In July 1973, the Head of Medical Services at the Ministry of Health, Dr. Preuss, wrote the Minister of Health that pathologists complied with the law and no longer used ‘blanco’ forms.^82^ However, a few months later, a hospital director wrote to the Ministry about a PME procedure used in hospitals, led by pathologists from the Hebrew University (Dr. James Mann) and its branch in Tel Aviv, the Greenberg Institute (headed by Dr. Heinrich Karplus).

According to the system we use, the Pathology Department sends the Ministry of Health PME forms with the names of the deceased and the family’s written consent to the hospital, and I, as a director, being requested to sign the forms. Dr. Mann and Dr. Karplus told me that the General Directors of the Health and Justice Ministries approved this arrangement. Nevertheless, obviously, this is fiction since I sign retroactively, having no knowledge about those cases. I ask for your guidance on this matter.^83^

After several years, these debates yielded, for the first time, criticism by representatives of secular-liberal circles in Israel. The example is Parliament Member Shulamit Aloni, head of a newly-founded civil-rights oriented Ratz party, in 1976. Aloni claimed that one had to distinguish between what the law permitted and what had to be done in cases without consent from the deceased or their families.

“Permitted” does not mean “must,” and if a person refuses to be operated on after their death, the law should turn “permission” into prohibition. One does not need to be religious and believe in the resurrection in order to be shocked by the Israeli authorities’ obtuseness to people’s right to their bodies and fate.^84^

The Attorney General answered Aloni’s remark eight months later, stating that since the cause of death could not be known in advance, the law could not determine whether to perform an autopsy before death.^85^

## Later Years: A Political Compromise

The 1977 elections led to a change of government in Israel. The coalitionary agreement between the Likud Party, now in power, and the AI included amending the Anatomy and Pathology Law. Contrary to previous governments led by Mapai and its secular allied parties, the Likud relied heavily on the NRP and AI for its newly-elected government. That was the main reason for the Likud’s consent to amend the law. The amendment stipulated that ‘PME would not be conducted without the family’s written consent. 'A person who has expressed his written opposition to PME would be honoured even if his family agreed to operate’. The amendment also referred to the dead with no family and stipulated they 'would not be operated on unless they had provided their written consent’.^86^

The heads of the IMA and Beilinson Hospital requested an urgent meeting with incoming Prime Minister Menachem Begin. In the meeting, they warned that ‘signing the agreement with the religious parties would get us below the red line and medicine would deteriorate’. They asked PM Begin to exclude medical emergencies from the amendment, in which doctors could operate. Begin claimed that the agreement’s details had already been signed and that he was committed to supporting it.^87^

After the formation of the government, a debate was held in parliament between Ora Namir of the opposition and the new Minister of Health, Eliezer Shostak. Namir warned that PME rates had dropped to 30 per cent, threatening the progress of medical science. On his part, Shostak defended the agreement with the ultraorthodox party and attacked the doctors, claiming that the amendments passed because doctors disregarded the law. Today, he added, PME rates ranged from 30 to 40 per cent, as customary in most developed countries, in which it was also customary to obtain the family’s consent.^88^ In a letter to Shostak, the IMA stated that they did not ignore the criticism of the State Comptroller and even condemned the blanco’ forms. However, ‘such an event does not justify changing the law’.^89^

We traced no ‘red line’ of PME rates in physicians’ internal correspondences and discussions between them and politicians throughout the 1970s. In addition, the data the two parliament members presented above were inconsistent with currently available data. According to Hoyert and Stempsey,^90^ in the late 1970s, national PME rates in the USA were 19 per cent and in early 1980s they dropped below 10 per cent. In Israel, in 1973, PME rates in government hospitals were 43 per cent—the nadir from 1956 to 1989. Connecting the ‘red line’ warning by IMA representatives and the seemingly inaccurate figures physicians presented was meant to pressure the new government to repeal the amendment. Nevertheless, Shostak was right about one thing: in the USA (as in Western Europe and China), PMEs required consent from the families of the deceased.^91^

Throughout 1980, Begin and Shostak received dozens of letters from the IMA, hospital directors and pathologists requesting to repeal the amendment. The chair of the Israel Psychiatric Society warned the Minister of Health that the amendments would halt the development of medical science and bring medical work back to ‘medieval conditions’. He even hinted that the psychiatric society would actively resist the law’s implementation. ‘If the law is passed in parliament, the psychiatric society will consider a policy per the interests of the patient and the public.’^92^

During the debates that preceded the vote, the parliament became a public battleground between politicians and physicians. Ultraorthodox cabinet members threatened to resign from the government, while the heads of the IMA threatened to deny medical treatment to parliament members intending to support the amendment, echoing the 1966 Revaha-Kaplan affair.^93^ In response, doctors were removed from the hall where the committee finalised the amendments, while secular politicians defended their presence. A day before the vote, IMA Chair Ram Ishay met the Prime Minister and the Minister of Health and threatened that the doctors would refuse to issue death certificates and even ‘stand trial and be punished if breaking the law is required to save lives’.^94^

The amendment passed narrowly by four votes on 2 December 1980. It seemed like the medical establishment lost its monopoly on doubt. However, in practice, the amendment included two exceptions demanded by secular parliament members (one of whom was Aloni) as a prerequisite for their support. First, a corpse was to be operated on either with the family’s consent or when no objection had been raised. Second, the body of individuals without a family would not be operated on unless they had consented to it in their lifetime. These provisions would not apply in the following cases: casualties of war and terrorist attacks, multi-casualty incidents or cases in which imminent danger to public health was identified; the Minister of Health was authorised to define what constituted ‘imminent danger’ to public health’.^95^ These provisions considered the wishes of the deceased and their family while excluding specific emergencies that would be determined by security and medical authorities.

The new stipulations to the amendments reproduced a medical monopoly under a new pretext. Two kinds of internal security became the basis of medical monopoly on PMEs. The first—a terrorist attack or mass accident, uses the language and tools of the military and police. The second, danger to public health, was determined by doctors: the relation between the dead and public health. In such cases, the rights of the deceased or their family are overruled by security mechanisms (army or police) or doctors. PME has turned into a medico-political matter by definition.

Moreover, as shown below, only well-organised groups (such as the ultraorthodox) supported by political parties (such as the AI) could now challenge the medical system. Indeed, the data show that PME rates in government hospitals increased a decade later. A 1990 survey by the Ministry of Health found that PME rates in the 1980s did not fall below the 1970s average and eventually increased by 10 per cent. In 1973, the national PME average was 43.3 per cent; in 1982, it rose to 44.4 per cent. In 1987, it increased again to 50.6 per cent; in 1989, it peaked at 54.8 per cent.^96^

After the amendment passed, three letters civilians sent to the Minister of Health can partly explain this trend. The first letter wondered about the state guarantee for individuals demanding that their bodies not be operated on after death. After all, nobody could control the time and place of their death, and their body could be taken to any hospital in Israel. Accordingly, the letter proposed that the Ministry of Health compile a list of citizens refusing PME. The Minister replied that this proposal was ‘not at all practical’.^97^ This shifted the onus in the struggle between the medical establishment, the law and society in Israel to citizens’ ability to organise to preserve their rights. It is likely that the relatively well-organised ultraorthodox Jews were more successful in this regard than others. A year later, another citizen wrote a similar letter to the Minister of Health. He asked how a hospital could know that a specific individual refused to be operated on after death. ‘After all, a person can go to different hospitals anywhere in the country in a situation in which he is not fully conscious.’^98^ His question remained unanswered.

In 1983, a rabbi, a representative of CSHD, wrote to the Director General of the Ministry of Health about the illegality of PMEs at Wolfson Hospital.^99^ The letter^100^ mentioned four cases in 1982 in which PMEs were performed in violation of the law. The doctors who performed the PME on a woman without her family's consent claimed that an autopsy was justified ‘due to a suspected infection, which may endanger other patients in the ward’. By contrast, the rabbi argued that no operation was needed to diagnose and prevent infection. Furthermore, the amended law stated clearly that in case of imminent danger to the public, such as mass infection, PME was allowed. However, the Ministry of Health had neither defined nor regulated the term ‘danger’ in other cases. How could PMEs be conducted, then? To the best of our knowledge, this letter remained unanswered as well.

## Conclusions

Since 1953, hospitals in Israel have become large-scale laboratories that provided corpses for pathological research. Through the broad interpretation of the formulation of the Anatomy and Pathology Law, which enabled almost unrestricted access to dead bodies, medical research thrived. Corpses became the raw materials through which the medical system produced scientific knowledge without requiring patients’ consent. When weighted against religious taboos and public resistance, doctors’ interests and vision had the upper hand for decades.

When medical discourse and practice are scrutinised, we find that doctors used uncertainty over the cause of death to justify PMEs that had no immediate clinical justification—against families’ wishes. The mass protest of two marginal Jewish groups—immigrants from Arab and Islamic countries and the ultraorthodox was followed by a temporary decline in PME rates in the late 1960s. However, the amendment to the law in 1980 changed little as PME rates resurged.

Aviad HaCohen suggests that the amendment ended the protest against the medical profession and removed the debate on PMEs from the public agenda since patients and their relatives could now decide on them.^101^ Indeed, protests against PMEs decreased from the late 1970s. However, rather than democratising PMEs, under the amended law, PMEs were now determined by security and medical authorities under an emergency, which they were authorised to define as such. The new law severely reduced the ability of any but organised groups, such as the ultraorthodox, to confront the medical profession, with limited effect at that.

Medical monopoly over the dead was determined for almost three decades, not so much by law as by the ability to maintain and cultivate scientific doubt. In this respect, monopoly was a product of the unethical creative endeavour, using a network of hospitals, the political and juridical systems and the press. Furthermore, doctors and the medical establishment utilised the modernist dichotomy of scientific progress as opposed to religious sentiment—a distinction that relied on the construction of the majority of protesters as ‘incited masses’. Doctors presented themselves as the spearhead of progress against religious traditionalism and reactionism. The distinction they produced (‘we’ pro-science vs. ‘they’, the non-scientific backward) created a dispute which concealed the fact that PMEs were conducted without informed consent. Finally, the reliance on PMEs required flexibility—turning one doubt (as to the cause of death) into another (concerning public health and security). The extent to which this affected PME rates is yet unknown.

## Footnotes

1 ‘Today—The Funeral of the Seven Accident Victims’, *Al Hamishmar*, 27 April 1965, 1 (Hebrew (H)).

2 Israeli State Archives (ISA), Ministry of Health—Anatomy and Pathology Law, C-17/6942, Letter to Kadish Luz (Parliament Chair), 1 July 1965.

3 These taboos have directly affected the status of Jewish medical students in Eastern and Central Europe. In early-20th century Austria and Poland, for example, non-Jewish students protested that although almost half of all medical students were Jewish, Jewish communities refused to authorise autopsies. Tensions around access to Jewish bodies were intertwined with antisemitism and allegations of Jewish ‘thirst’ for Gentile corpses. Tatjana Buklijas, ‘Cultures of Death and Politics of Corpse Supply: Anatomy in Vienna, 1848–1914’, *Bulletin of the History of Medicine*, 2008, 82, 570–607; Natalia Aleksiun, ‘Jewish Students and Christian Corpses in Interwar Poland: Playing with the Language of Blood Libel’, *Jewish History*, 2012, 26, 327–42.

4 Lester King and Marjorie Meehan, ‘A History of Pathology’, *American Journal of Pathology*, 1973, 73, 513–44.

5 King and Meehan, ‘A History of Pathology’; Jacek Gulczyński, Ewa Iżycka-Świeszewska, and Marek Grzybiak, ‘Short History of the Autopsy: From the Second Half of the 16th Century to Contemporary Times’, *Polish Journal of Pathology*, 2010, 3, 169–75.

6 Zhu Ming-hua and Yu Dang-hui, ‘Medical Progress: Fluctuations in the Rate of Autopsy in China’, *Chinese Medical Journal*, 2011, 124, 3404–407; Kristen Adams, ‘The Usefulness of Autopsy’, *Laboratory Medicine*, 2020, 51, e42–44.

7 William Stempsey, ‘The Penetrating Gaze and the Decline of the Autopsy’, *AMA Journal of Ethics*, 2016, 18, 833–38; Donna Hoyert, ‘The Changing Profile of Autopsied Deaths in the United States, 1972–2007’, *NCHS Data Brief*, 2016, 67, 1–8.

8 Kaveh Shojania and Elizabeth Burton, ‘The Vanishing Nonforensic Autopsy’, *New England Journal of Medicine*, 2008, 358, 873–75.

9 Aviad HaCohen, ‘Law and Medicine in a “Jewish and Democratic State”: Between Anatomy of Law and Pathology of State–Religion Relationships’, *Shaare’ Mishpat*, 2000, 2, 189–221 (H); Ron Rot, ‘Bodies Insulted—Shaming and Honor in the Struggle of Post-Mortem Examinations in Israel’, *Law and Business*, 2020, 23, 1–55.

10 Michel Foucault, *Territory, Security, Population: Lectures at the Collège de France, 1977–78* (Basingstoke, UK: Palgrave Macmillan, 2007), 311–32; Idem., *Society Must Be Defended: Lectures at the Collège de France, 1975–76* (New York: Picador, 1997), 239–64. See also Yehouda Shenhav, ‘Imperialism, Exceptionalism, and the Contemporary World’, in Marcelo Svirsky and Simone Bignall, eds, *Agamben and Colonialism* (Edinburgh: Edinburgh University Press, 2012), 17–32.

11 Foucault, *Society Must Be Defended, 248*.

12 Khaled Fahmy, *In Quest of Justice: Islamic Law and Forensic Medicine in Modern Egypt* (Berkeley: University of California Press, 2018), 62–77.

13 Shmuel Berkovitz, ‘Legal Aspects of PMEs in Israel’, *Dinei Israel*, 1977, 65, 143–81 (H).

14 For a discussion of the nature and implications of anatomy classes at the Hebrew University, see Liat Kozma and Benny Nuriely (forthcoming), ‘The Contribution of the Dead to Anatomy Classes at the Hebrew University Medical School, 1950–1967’, *Zmanim: A Historical Quarterly* (H).

15 Emanuel Gutman, ‘The Religious Cleavage’, in Moshe Lissak and Baruch Knei-Paz, eds, *Israel Toward the New Millennium: Society, Politics, and Culture* (Hebrew University Press, 1996), 61–73.

16 See LIat Kozma and Benny Nuriely (forthcoming)' Aristocracy of Sorts”: The Foundation of the Hebrew University Medical School, 1949-1967, Ifat Weiss and Uzi Ravhun (eds.), The Genealogy of the Hebrew University in Jerusalem .

17 Gutman, ‘The Religious Cleavage’, 1996.

18 ISA, Ministry of Health—Anatomy and Pathology Law, Volume 1, C-1/5084, Chief Rabbinate to Minister of Health Moshe Shapira; Dr. Chaim Sheba to Moshe Shapira, 27 February 1951.

19
*Ibid*., 8 May 1951.

20 HaCohen, ‘Law and Medicine’, 196.

21 ISA, Ministry of Health—Anatomy and Pathology Law, Volume 1, C-1/5084—Public Service Committee, 11 August 1953, 9.

22 Anatomy and Pathology Law (1953), Israeli Law Book, 134, 4 September 1953.

23 ISA, Ministry of Health—Anatomy and Pathology Law, Volume 2, C-2/5084, script from ‘Hamodia’ daily, 8 March 1954.

24 ISA, Ministry of Health—PMEs for Research and Determining the Cause of Death, C-14/5083, script from a letter by a resident of Shaar Menashe Institution, undated document.

25 ISA, Ministry of Health—PMEs for Research and Determining the Cause of Death, C-14/5083, Mr. Shlomo Adani (Religious Council, Rosh HaAyin) to Ministry of Health, 12 August 1954.

26 ISA, Ministry of Health—Anatomy and Pathology Law, Volume 2, C-2/5084, Zerah Varhaftig to Minister of Health, 19 January 1954; Dr. Gjebin to Minister of Health, 24 February 1954.

27
*Ibid*., Minister of Health Yosef Serlin to Deputy Minister of Religions, 31 March 1954.

28
*Ibid*., Dr. Gjebin to Minister of Health on PMEs, 2 April 1954.

29
*Ibid*., Varhaftig to Minister of Health, 2 April 1954, 19 April 1954, 13 May 1954; Varhaftig to Attorney General, 18 August 1954.

30
*Ibid*., Minister of Health Serlin to Ministry of Health General Director, 2 September 1954.

31 ISA, Ministry of Health—PMEs for Research and Determining the Cause of Death, C-14/5083, Dr. Batish to Dr. Meir and Dr. Friedenthal, ‘Commission of Inquiry into the Implementation of the Anatomy and Pathology Law’, 17 September 1954.

32 ISA, Ministry of Health, Anatomy and Pathology Law, Volume 2, C-2/5084, ‘Inquiry Commission’s Report on the Implementation of the Anatomy and Pathology Law’, undated document.

33
*Ibid*., Dr. Meir to Ministry of Health, 23 November 1955.

34
*Ibid*., Dr. Meir to Ministry of Health’s General Director, 13 January 1956.

35 ISA, Ministry of Health, PMEs for Research and Determining the Cause of Death, C-14/5083, Dr. Bathish to hospital administrations, 15 February 1956.

36
*Ibid*., H. S. Halevi, ‘Memorandum on the Issue of PMEs in Israeli Hospitals’, 30 December 1957.

37 ISA, Ministry of Health, GL-14/2088, PMEs for Research and Determining the Cause of Death. Mortality Survey at the Tel HaShomer Hospital, 1961–62.

38 Shojania and Burton, ‘The Vanishing Nonforensic Autopsy’, 874; Adams, ‘The Usefulness of Autopsy’, e44.

39 Nurit Kirsh, ‘Genetic Research on Israel’s Populations: Two Opposite Tendencies’, in Volker Roelcke and Giovanni Maio, eds, *Twentieth Century Ethics of Human Subjects Research: Historical Perspectives on Values, Practices, and Regulations* (Stuttgart: Steiner, 2004), 310–11; Nurit Kirsh and Ari Barel, ‘The Hospital as a Laboratory: Population Studies at Tel HaShomer Hospital in Israel, 1950s–1960s’, *Science in Context* (forthcoming).

40 ISA, Ministry of Health—PMEs for Research and Determining the Cause of Death, C-14/5083, Itzhak Levin, Shlomo Lorincz and Shlomo Gross to Minister of Health, 10 May 1959.

41
*Ibid*., letter to Zerah Varhaftig, sender unknown, 8 October 1959.

42
*Ibid*., letter by Tawila Mansour (El Tira), 9 February 1959; Dr. Hecht to Dr. Brachot, 25 March 1959.

43 ISA, Ministry of Health, Anatomy and Pathology Law, Vol. 2, C-2/5084, ‘Conclusions of the Constitution, Law and Justice Committee on PMEs’, 13 October 1959.

44 ISA, Ministry of Health—PMEs for Research and Determining the Cause of Death, C-14/5083, Dr. Meir to Minister of Health, 15 June 1959.

45
*Ibid*., Dr. Naor (Kaplan Hospital) to Dr. Meir, 16 April 1959.

46 For example, according to Director of Rambam Hospital in Haifa, Director of the Greenberg Pathological Institute in Tel Aviv Dr. Heinrich Karplus asked him to sign a blank autopsy form before the patient passed away. ISA, PMEs for Research and Determining the Cause of Death, GL-15/12185, Dr. Preuss to Minister of Health, 9 July 1973.

47 ISA, Ministry of Health—PMEs for Research and Determining the Cause of Death, C-15/5083, letter to Dr. Mann (Hadassah Hospital), 5 January 1961; letter by Dr. Meir, 27 February 1961.

48
*Ibid*., letter by lawyer Z. HaCohen to Dr. Mann, 9 February 1961.

49
*Ibid*., letter to the Ministry of Health, 10 June 1961.

50 ISA, Prime Minister Office, Anatomy and Pathology Law, C-17/6942, Prof. Unger and Dr. Birenbaum, 28 November 1961.

51
*Ibid*., Dr. Kaplinski and Prof. Adler (IMA), ‘Memorandum Anatomy and Pathology Law’, 1 July 1965.

52
*Ibid*., ‘The new Anatomy and Pathology bill undermines medicine’, undated.

53
*Ibid*., Yigal Alon to Government Secretary, 28 July 1965.

54 ‘Internal Organs Removed from Deceased Body from Revaha’, *HaTzofe*, 23 May 1966, 5 (H).

55 ‘Kaplan Doctors Refused to Treat Patients from Revaha’, *Maariv*, 25 March 1966, 23 (H).

56 ‘Parliament Discusses Revaha People against Kaplan Doctors’, *Davar*, 2 June 1966, 2 (H).

57 ‘Kaplan Hospital Renews Treatment to Revaha Residents’, *HaTzofe*, 20 June 1966, 6 (H).

58 ‘Indictment against Revaha Residents’, *Lamerhav*, 23 June 1966, 5 (H).

59 ‘6 Residents of Revaha Imprisoned for Rioting in Hospital’, *Maariv*, 9 March 1967, 7 (H).

60 For example, see HaCohen ‘Law and Medicine; Rot, ‘Bodies Insulted'.

61 ‘Mass Rallies against PMEs’, *Maariv*, 16 August 1966, 6 (H).

62 ‘Police Self-Restraint Prevented Mass Riot in the HaTikva Neighborhood’, *Maariv*, 15 February 1967, 3 (H).

63 ‘Rally against PMEs in Rishon LeZion’, *Lamerhav*, 7 March 1967, 2 (H).

64 ‘Violent Demonstration Against PMEs in the Capital; 30 Injured’, *Al HaMishmar*, 15 March 1967, 3 (H).

65 For instance, letter by CSHD to Director of Assaf HaRofeh Hospital, 10 October 1967.

66 Shmuel Berkovitz, ‘Legal Aspects of PMEs in Israel’, *Dinei Israel*, 1977, 65, 143–81 (H).

67 ISA, Ministry of Health, GL-14/2088, PMEs for Research and Determining the Cause of Death, 10 April 1967.

68
*Ibid*., Prof. Adler to Prof. Liben, 5 April 1967.

69
*Ibid*., Ministry of Health to Dr. Sheba, 12 May 1967.

70
*Ibid*., Dr. Sheba to Dr. Brachot, 16 May 1967.

71
*Ibid*., Sheba to Ministry of Health, 8 August 1967.

72
*Ibid*., Dr. Stein to Minister of Health, 21 December 1967.

73 Parliament Meeting, Motion for the Agenda, no. 846—The Secret Protocol of Hospital Directors on PMEs, 13 November 1968.

74 Adler referred to pathological examinations performed in governmental hospitals rather than anatomic dissection performed in anatomy classes. The subject of anatomic dissection is beyond the scope of this article. For further reading on this topic, see Liat Kozma and Benny Nuriely (forthcoming) ‘The Contribution of the Dead to Anatomy Classes at the Hebrew University Medical School, 1950–1967’, *Zmanim: A Historical Quarterly* (H).

75 State Comptroller, Annual Report No. 19, 1968; published on 14 February 1969.

76 For example, in a letter to Dr. Karplus (head of the Greenberg Pathological Institute in Tel Aviv), Gjebin wrote, ‘Your attention again to the Anatomy and Pathology Law. By law, you are instructed to perform an autopsy only with a certificate signed by three doctors […] you are requested to sign the monthly report that PMEs were indeed performed according to the law’, ISA, GL-14/12185, Post-mortem Operations, Gjebin to Karplus, 10 April 1969.

77 ISA, PMEs for Research and Determining the Cause of Death, GL-16/2088, 9 July 1969.

78 ISA, Anatomy and Pathology Law, C-17/6942, 13 August 1969.

79
*Ibid*., Minister of Health to Government Secretary, 23 September 1969.

80 ISA, PMEs for Research and Determining the Cause of Death, GL-16/2088, Ministry of Health to Health Bureau, Ramle County, 25 September 1969. Emphasis in the original.

81
*Ibid*., P. Dagan to Director General of Ministry of Health, 1 July 1969.

82 ISA, PMEs for Research and Determining the Cause of Death, GL-15/12185, Dr. Preuss to Minister of Health, 9 July 1973.

83
*Ibid*., Dr. M. Ravid to Ministry of Health, 11 January 1974.

84
*Ibid*., Shulamit Aloni to Attorney General Aharon Barak, 4 March 1976.

85
*Ibid*., Attorney General to Shulamit Aloni, 19 November 1976.

86 ISA, Anatomy and Pathology Law and Determining Clinical Death, GL-4/14/41028.

87 ‘Begin Rejects Doctors’ Demand to Remove Restrictions on PMEs’, *Maariv*, 17 June 1977, 4 (H).

88 ‘Hospitals Kept Pre-Signed Forms by Doctors for PMEs’, *Maariv*, 27 July 1977, 6 (H).

89 ISA, Ministry of Health, GL-19/12185, IMA to Minister of Health, 31 July 1977.

90 Hoyert, ‘The Changing Profile’, 5; Stempsey, ‘The Penetrating Gaze’, 835.

91 Ming-hua and Dang-hui, ‘Medical Progress’, 3,406; Gulczyński, Świeszewska, and Grzybiak, ‘Short History of the Autopsy’, 174; Stempsey, ‘The Penetrating Gaze’, 836.

92 ISA, Anatomy and Pathology Law and Determining Clinical Death, GL-4/14/41028, Prof. Ashael to Minister of Health, 30 November 1980. Deaths of psychiatric patients in governmental and private institutions were to be investigated by a coroner, according to the Cause of Death Investigation Law (1958), which dealt mainly with criminal investigations. This law is beyond the scope of this paper, and the inclusion of mental patients here was probably designed to investigate cases of ill-treatment and neglect in mental hospitals. Psychiatrists here might have feared that the inclusion of consent here would prevent the detection of such cases.

93 ‘Charlatans, Called out Lorincz, We Will not Treat You—the Doctors Replied in Anger’, *Maariv*, 27 November 1980, 3 (H).

94 ‘Doctors: Without PME, No Death Certificates Will be Issued’, *Maariv*, 1 December 1980, 2 (H).

95 ISA, Anatomy and Pathology Law and Determining Clinical Death, GL-14/41028.

96 ISA, Ministry of Health, Anatomy and Pathology (special cases), GL-13/23840.

97 ISA, Anatomy and Pathology Law and Determining Clinical Death, GL-4/14/41028, letter by the Minister of Health to John Doe, 19 August 1981.

98
*Ibid*., Natan Bram to Minister of Health, October 1981.

99 Located in the city of Holon, Wolfson Hospital was founded in 1980 following the growth in population south of Tel Aviv. Initially, Wolfson was made up of several departments moved from the old Donolo and Dajani hospitals in Jaffa and Shmuel HaRofeh and Assaf HaRofeh hospitals. Haim Zacut, ‘Resurrection of a Skeleton: Founding the Edith Wolfson Hospital in Holon’, *HaRefuah*, 2018, 157, 398–400 (H).

100 ISA, Anatomy and Pathology Law and Determining Clinical Death, GL-4/14/41028, Rabbi Winer to Prof. Baruch Modan, 11 March 1983.

101 HaCohen, ‘Law and Medicine’, 206.

